# Income, food expenditure shares, and severe food insecurity in Australia across 21 waves of HILDA

**DOI:** 10.1093/heapro/daag079

**Published:** 2026-06-04

**Authors:** Alfredo R Paloyo, Katherine Kent, Alemayehu Gebremariam, Karen Charlton

**Affiliations:** School of Business, Faculty of Arts, Society, and Business, University of Wollongong, 2 Northfields Avenue, Keiraville, NSW 2500, Australia; School of Medical, Indigenous, and Health Sciences, Faculty of Science, Medicine, and Health, University of Wollongong, 2 Northfields Avenue, Keiraville, NSW 2500, Australia; School of Medical, Indigenous, and Health Sciences, Faculty of Science, Medicine, and Health, University of Wollongong, 2 Northfields Avenue, Keiraville, NSW 2500, Australia; School of Health Sciences, College of Health, Medicine, and Wellbeing, University of Newcastle, University Drive, Callaghan, NSW 2308, Australia; Nutrition and Metabolic Health Program, Hunter Medical Research Institute, Lot 1, Kookaburra Cct, New Lambton, NSW 2305, Australia

**Keywords:** food insecurity, health equity, social determinants of health, income inequality, Australia

## Abstract

This study aimed to examine the association between household income and severe food insecurity in Australia and to assess how income-related patterns vary across geographic scales. Using 21 waves (2001–2021) of the Household, Income, and Labour Dynamics in Australia (HILDA) survey, we measured severe food insecurity through reported meal skipping due to financial constraint and food-related economic pressure through the share of household income spent on food. All estimates were weighted using HILDA population weights matched to each outcome. At the national level, weighted prevalence of meal skipping was 3.6% over the pooled sample (3.4% in the most recent wave), with a pronounced income gradient: individuals in the lowest income percentiles had a predicted probability of meal skipping exceeding 7%, compared with <1% at the 95th percentile. Food expenditure share declined monotonically with rising income, with households in the lowest quintile spending more than 30% of equivalized income on food. Among those who ever reported meal skipping in the balanced panel, the experience was episodic for most (5.8% of respondents reported it in exactly one of 21 waves) but recurrent for a nontrivial minority (9.1% in two or more waves). We formally tested whether prevalence in the Illawarra Shoalhaven region differed from the national average and found no statistically significant difference (*F* = 1.01, *P* = 0.315), suggesting that the income-gradient finding generalizes across geographic contexts. Severe food insecurity in Australia is strongly and persistently patterned by income, reinforcing income adequacy as the central policy lever for reducing hunger in high-income settings.

Contribution to Health Promotion

- Two decades of nationally representative longitudinal data show a strong income gradient in severe food insecurity measured through meal skipping.
- Income gradient generalizes across geographic contexts, with no evident regional differences using the Illawarra Shoalhaven as a case study.
- Provides population-level evidence to support upstream, equity-focused health promotion responses that address income adequacy and cost-of-living pressures.

## Introduction

Food insecurity, defined as the lack of reliable access to adequate, nutritious food for an active and healthy life (Food and Agriculture Organisation of the United Nations 2012), is an escalating concern in high-income nations, including Australia (Pollard and Booth 2019). Food insecurity is multidimensional, spanning six inter-related pillars: food availability, accessibility, utilization, stability, agency, and sustainability (Clapp *et al*. 2022). These dimensions reflect not only whether food exists in the food supply system but also whether individuals can physically and economically access it, transform it into meals using available resources, make autonomous decisions about their food, and rely on a food system that is stable and ecologically viable over time (Clapp *et al*. 2022). Given the relatively abundant and accessible food supply in high-income contexts, however, affordability and income adequacy are consistently identified as the strongest correlates shaping household food security in these settings.

Recent national estimates from the Australian Bureau of Statistics indicate that 13.2% of Australian households experienced food insecurity in 2023, including 8.5% experiencing moderate or severe food insecurity (ABS 2025). Notably, this represents the first nationally representative, multi-item estimate of household food insecurity released by the ABS in more than a decade, following a prolonged absence of routine national surveillance. The ABS findings document a socioeconomic gradient, with food insecurity most prevalent among low-income households, and other groups with lower incomes such as lone-parent families, and people living in group households. It has been argued that food insecurity has become more widespread in the wake of rising living costs, stagnant wages, and the economic shocks of the COVID-19 pandemic and subsequent inflation (Botha and Payne 2022). Evidence also shows that food insecurity rarely occurs in isolation, but that it frequently co-exists with financial stress, poor health, and housing insecurity (Botha *et al*. 2024), with households often resorting to increasingly severe coping strategies such as cutting insurance or forgoing medical care (Seivwright *et al*. 2024). Together, this evidence underscores income adequacy as a central correlate of severe food insecurity in Australia.

Despite these advances in national surveillance, longitudinal monitoring of food insecurity and its relationship with income remains limited. The Household, Income and Labour Dynamics in Australia (HILDA) survey provides an important complementary data source through its long-standing measure of severe food insecurity (skipping meals) due to financial constraints in the previous 12 months. While this indicator captures only the most acute manifestation of food insecurity, it offers a conservative and policy-relevant marker of economic hardship over time (Harttgen and Seiler 2023). HILDA also includes detailed income and expenditure data, enabling assessment of the economic burden associated with food access. In particular, examining the share of household income spent on food provides insight into financial strain and adaptive behaviours, such as reduced dietary quality, budget reallocation, or reliance on informal and charitable food sources, which may precede overt indicators such as meal skipping (Lewis *et al*. 2023, Garratt and Armstrong 2024).

Understanding how income-related food insecurity varies not only over time but also across geographic contexts is critical for informing policy responses. National estimates, while essential for benchmarking and monitoring trends, can obscure substantial regional variation driven by differences in housing costs, labour markets, food environments, and access to services (Foodbank Australia 2023). This distinction is particularly salient in Australia, where many determinants of food insecurity, including housing, health services, planning, and aspects of social support, are shaped at state and local levels (Yii *et al*. 2020). Local studies have sought to address this gap by documenting food insecurity prevalence and food environment characteristics within specific regions (Kent 2025). However, most lack the representativeness, statistical power, or longitudinal scope required to assess trends or situate local conditions within broader national income gradients.

This tension motivates our inclusion of the Illawarra Shoalhaven region of New South Wales (a coastal region ∼80 km south of Sydney, population ∼400 000) as a regional case study, where multiple sources of local evidence have indicated food-related vulnerability but where the relationship to national income-driven patterns has not been formally tested. A 2024 regional survey of over 600 households found that 38% were food insecure using the 18-item USDA Household Food Security Survey Module, with children affected in nearly one-third of families with dependents (Kent *et al*. 2025). Food insecurity was strongly associated with poor mental and physical health, diagnosed mental illness, and social isolation. Community organizations have reported unprecedented demand for food relief, particularly among first-time users in the context of rising living costs (Cartwright 2025). Additional pressures arise from an unhealthy food environment, characterized by an unhealthy-to-healthy food outlet ratio of approximately six-to-one, and the high cost of a basic healthy diet, estimated to exceed 25% of disposable income for many welfare-dependent households (Charlton *et al*. 2024, Fishlock *et al*. 2025).

What remains underexplored is how national food insecurity data can be leveraged to assess income-related vulnerability across geographic scales and over time. This study addresses this gap by examining food insecurity at national, state, and regional levels using longitudinal data from the HILDA survey. We focus on two complementary indicators: severe food insecurity, measured by meal skipping due to financial constraint, and the economic burden of food access, assessed through household food expenditure share.

The objectives of this study are to:

1. Examine how household income gradients are associated with severe food insecurity and the economic burden of food access in Australia.
2. Describe national, state, and regional patterns in severe food insecurity over time, and formally test whether prevalence in the Illawarra Shoalhaven region of New South Wales differs from the national average.

By integrating national longitudinal data with a regional case study, this research aims to improve interpretation of population-level food insecurity monitoring and contribute evidence to support more responsive, place-sensitive policy approaches in Australia and other high-income settings.

## Methodology

### Data source, sample, and ethics

This study used Waves 1–21 (2001–2021) of the HILDA survey, a nationally representative longitudinal panel study of Australian households that collects annual data on income, employment, wellbeing, and household expenditure. We restrict the analysis to individuals aged 15 years and older. The resulting dataset is an unbalanced panel with (Individual × Wave) = 335 600, with the number of individuals per wave ranging from 13 037 to 18 433. This study uses confidentialized unit record data from the HILDA Survey, which is conducted by the Melbourne Institute at the University of Melbourne and is funded by the Australian Government Department of Social Services. The data are made available through the Australian Data Archive. As the data are de-identified and collected under existing ethical approvals pertinent to HILDA, no additional institutional ethics approval was required for this study.

### Study setting and geographic scale

Analyses were undertaken at multiple geographic scales (national, state/territory, and a regional case study). The Illawarra Shoalhaven analysis serves an exploratory, hypothesis-generating purpose rather than providing definitive regional estimates. Given small sample sizes, year-to-year variation likely reflects sampling volatility as much as genuine trends. These results are best interpreted as suggestive patterns that motivate dedicated regional surveys rather than as standalone evidence of divergence from national trends. National estimates were produced using the full HILDA sample. State- and territory-level estimates were generated using HILDA jurisdiction identifiers for respondents’ place of residence at the time of interview. To examine subnational patterns in more detail, we used the Illawarra Shoalhaven region of New South Wales (a coastal region in New South Wales ∼80 km south of Sydney, with a population of roughly 400 000) as a regional case study. The region includes the local government areas of Wollongong, Shellharbour, Kiama, and Shoalhaven. To identify the Illawarra Shoalhaven region within the HILDA dataset, we used Statistical Area Level 2 (SA2), the second tier of the Australian Bureau of Statistics geographic classification (yielded 37 SA2 codes in total). Residence was treated as time-varying, such that respondents contributed to the Illawarra Shoalhaven series in waves where they resided in the relevant SA2 s. The Illawarra Shoalhaven subsample ranged from 284 to 358 individuals across waves.

## Measures

### Severe food insecurity

We employ two types of food insecurity measures from HILDA: skipping a meal because of a lack of funds and household food expenditure share. Our primary indicator is derived from the yes/no survey item asking whether the respondent ‘went without meals in the past 12 months because of a shortage of money’. This measure captures severe food insecurity—effectively, episodes of hunger caused by lack of funds. It corresponds to one of the more extreme items in the Food Insecurity Experience Scale. We code the respondent as food insecure under this measure if they answer ‘yes’ to skipping meals for financial reasons. This binary variable serves as the dependent variable in our analysis of prevalence and correlates of food insecurity. While this measure underestimates milder forms of food stress, it has the advantage of clearly identifying households with acute food shortages. Health is self-reported and weight categories are based on BMI, which are themselves based on self-reported height and weight and are not objective measured.

### Food-related economic pressure (food expenditure share)

To account for the household size (i.e. the number of household members) and the ages of its members, we calculate the equivalized household income by dividing annual household income by the following equivalence scale:


$$es_{h}=1+0.5\times (a_{h}-1)+0.3\times (c_{h}),$$

where $es_{h}$ is the equivalence scale, $a_{h}$ is the number of adults, and $c_{h}$ is the number of dependent children in household *h*.

As an economic measure related to food security, we calculate the share of household income spent on food. HILDA collects data on household expenditures, including an aggregate of weekly spending on groceries and eating out (nondurable consumption). We derive each household’s food expenditure share as


$$fes_{h}=\frac{\;fs_{h}}{y_{h}}\times 100\text{\%},$$

where $fes_{h}$ is the food expenditure share of household *h*, $fs_{h}$ is the annual food expenditure of household *h*, and $y_{h}$ is the annual equivalized household income of household *h*. We restricted the sample to non-negative income values and truncated at $300 000 to minimize the influence of extreme values on predicted probabilities. Since food expenditure in HILDA is measured weekly, we derive an annual measure by multiplying the reported weekly expenditure by 52. We acknowledge that annualizing weekly food expenditure assumes uniform spending patterns throughout the year, likely introducing measurement error. Ideally, monthly or quarterly expenditure data would reduce this bias. However, HILDA provides only weekly measures.

The variable $fes_{h}$ is an indicator, which reflects the budgetary burden of food. A higher share means a household is spending a larger portion of its income to meet food needs, which can signal economic vulnerability and potential food stress and food insecurity. Internationally, the food expenditure share is a recognized proxy for food security (Data4Diets 2023): poorer households tend to spend a greater proportion of their income on food, and exceptionally high food share percentages are often associated with food insecurity. We use this measure to predict the likelihood of skipping meals.

### Analytical approach

All statistical analyses were conducted using Stata/SE 18.0. Access to the Household Income and Labour Dynamics in Australia (HILDA) dataset was provided under a confidential licencing agreement. Analyses were structured around two objectives: (1) national income gradients and economic burden; and (2) national, state/territory, and regional patterns in severe food insecurity over time.

All estimates in this study use HILDA population weights matched to the relevant outcome. Prevalence statistics and regression models with outcomes drawn from the Self-Completion Questionnaire (SCQ), including meal skipping, are weighted using the SCQ responding-person weight (hhwtsc). Outcomes drawn from the Person Questionnaire (PQ), including income and employment variables, are weighted using the PQ responding-person weight (hhwtrp). Household expenditure variables, which are collected via the Household Questionnaire, are similarly weighted using hhwtrp. Longitudinal analyses of repeat reporting use the longitudinal weight (lnwtrp). The survey design is declared with stratification on the HILDA stratum variable (xhhstrat), which accounts for the stratified sampling design of the original HILDA sample.

Standard errors in all pooled multiwave regressions are clustered on the persistent person identifier (xwaveid), which accounts for repeated observations of the same individual across waves. In HILDA, household identifiers (hhrhid) are wave-specific and change when household composition changes, making them unsuitable for clustering in pooled longitudinal models. For single-wave analyses, standard errors are clustered at the household level using the wave-specific household identifier.

Economic burden of food access (Objective 1): We examined the relationship between equivalized household income and food expenditure share using Engel curves. Engel’s law states that individuals with lower equivalized incomes spend a substantially higher proportion of their available resources on food (Data4Diets 2023), and this is seen as a downward-sloping curve. We employed locally weighted scatterplot smoothing (LOWESS) to explore nonlinear relationships and to visually represent the income–expenditure share gradient. The Engel curve specifications include month-of-interview fixed effects to account for seasonal variation in food expenditure patterns, as food spending may systematically vary across the year due to holiday periods, seasonal price fluctuations, and weather-related consumption changes.

Temporal and geographic comparisons (Objective 2): We produced descriptive trend figures showing the annual prevalence of meal skipping nationally (2001–2021), disaggregated by state and territory, and for the Illawarra Shoalhaven as a regional subsample. These comparisons were used to assess whether regional patterns broadly track national trends or whether periods of divergence are observed.

A key limitation of our analytical approach arises from constraints imposed by sample size in the regional subsample. Specifically, the Illawarra Shoalhaven subsample contains a relatively small number of respondents per wave, limiting statistical power and the precision of estimated relationships. Consequently, caution should be exercised in interpreting regional-level findings for the Illawarra Shoalhaven, as estimates may be sensitive to sample composition and may not be broadly generalizable. Accordingly, the regional results are interpreted as indicative patterns suitable for triangulation with dedicated regional surveys and complementary data sources.

## Results

### National income gradients and the economic burden of food access

**Figure 1:**
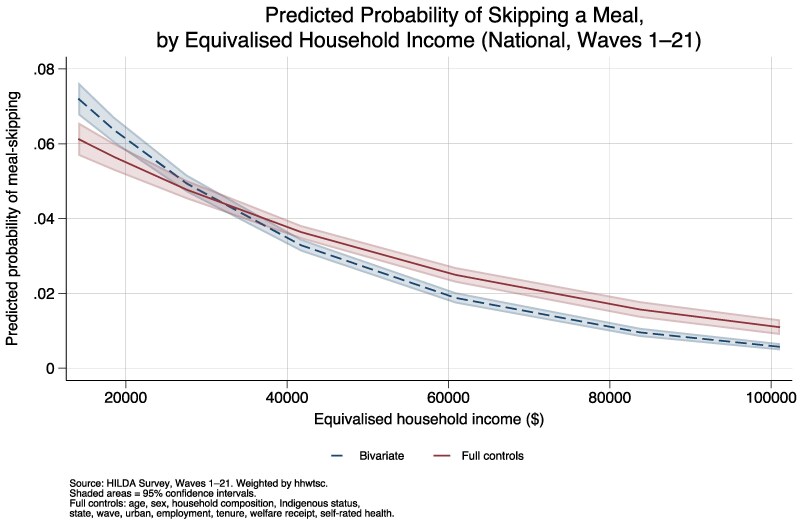
Predicted probability of skipping a meal due to financial constraint across the income distribution (Australia, waves 1–21).

The relationship between equivalized household income and the predicted probability of meal skipping, estimated using weighted logistic regression at the national level, is presented in Fig. 1. The figure shows both a bivariate specification (income only) and a full specification controlling for age, sex, household composition, Indigenous status, state, wave, urban/rural residence, employment status, housing tenure, welfare receipt, and self-assessed health. In the bivariate model, individuals at the 5th percentile of equivalized income ($14 120) had a predicted probability of meal skipping of 7.2% (95% CI: 6.8%–7.6%), declining to 0.6% (95% CI: 0.5%–0.7%) at the 95th percentile ($101 083). After adjusting for the full set of controls, the gradient attenuated modestly. The predicted probability at the 5th percentile was 6.1% (95% CI: 5.7%–6.6%) and 1.1% (95% CI: 0.9%–1.3%) at the 95th percentile but remained strong and statistically significant (average marginal effect of equivalized income: −0.73 percentage points per $10 000 increase, *P* < 0.001).

Supplementary Table S6 presents the full logistic regression results for meal skipping across four specifications. In the bivariate model (Column 1), a $10 000 increase in equivalized household income was associated with a reduction of 0.30 log-odds units in the probability of meal skipping (*P* < 0.001). Adding demographic controls (Column 2: age, sex, household composition, Indigenous status, state, wave, urban/rural) strengthened the income coefficient to −0.43 log-odds units per $10 000 (*P* < 0.001), reflecting confounding between income and demographic characteristics. The full specification (Column 3) added employment status, housing tenure, welfare receipt, and self-assessed health; the income coefficient attenuated to −0.22 log-odds units per $10 000 (*P* < 0.001), indicating that some of the income effect operates through these mediating pathways. Because equivalized income is measured in annual dollars, the per-dollar coefficient is very small (on the order of −0.00002) and appears as −0.000 in Supplementary Table S6 due to three-decimal-place formatting; the per-$10 000 figures reported here are derived from full-precision estimates in the Stata log files. Welfare receipt was strongly associated with meal skipping (coefficient: 0.72, *P* < 0.001), as were renting (0.76, *P* < 0.001) and unemployment (0.73, *P* < 0.001). In the COVID sensitivity specification (Column 4), income–COVID interaction terms were not statistically significant (*P* = 0.200 and *P* = 0.959 for 2020 and 2021, respectively), indicating that the income gradient did not change meaningfully during the pandemic years.

Supplementary Table S7 presents Engel curve OLS regressions. In the bivariate Wave 21 specification, a one-unit increase in log equivalized income was associated with a 14.6 percentage-point reduction in the food expenditure share (*β* = −14.571, *P* < 0.001, *R*^2^ = 0.33). Adding demographic controls and month-of-interview fixed effects left the income coefficient virtually unchanged. The pooled all-waves specification yielded a coefficient of −16.6 percentage points (*P* < 0.001), confirming the stability of this relationship across time.

**Figure 2:**
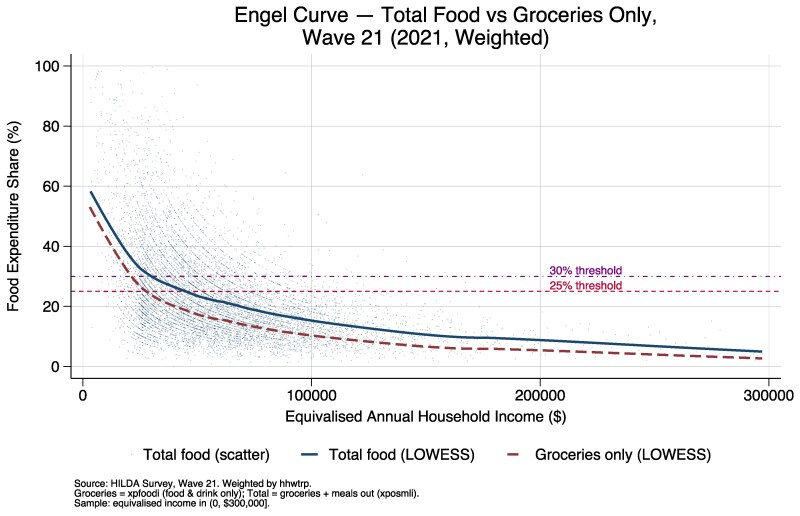
Engel curve showing household food expenditure share (%) by equivalized household income (Australia, 2021).

Figure 2 presents updated Engel curves for Australia in 2021, illustrating the relationship between equivalized household income and the share of income spent on food under two definitions: total food expenditure (groceries plus meals eaten outside the home) and groceries only. Both definitions show the expected downward-sloping relationship consistent with Engel’s law. Under the total food definition, 4774 households (27.4% of the Wave 21 sample) spent more than 30% of their equivalized income on food. Under the groceries-only definition, this figure was 2582 households (14.8%), indicating that roughly half of the households exceeding the 30% threshold did so partly because of expenditure on meals eaten outside the home. The claim that some households allocate more than half their income to food survives income trimming: after excluding households with equivalized income below $5000 per year, 13 681 households in the pooled sample still exceeded the 50% threshold, compared with 15 184 in the untrimmed sample. The groceries-only Engel curve provides a more conservative picture of food-related financial pressure but does not eliminate the finding that low-income households face a substantial economic burden in meeting food needs.

### National, state, and regional patterns of severe food insecurity

**Figure 3:**
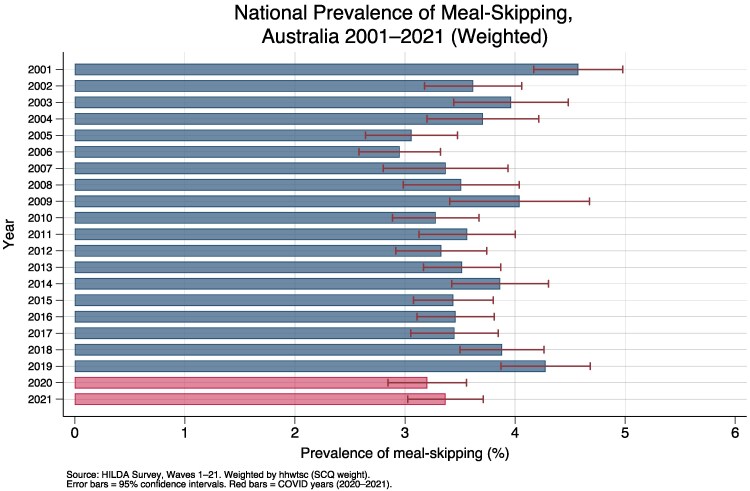
National prevalence of meal-skipping, Australia 2001–2021.

The weighted national prevalence of meal skipping is 3.6% (pooled) compared with the 3.9% unweighted prevalence (difference of 0.31 percentage points). Supplementary Table S8 presents summary statistics for selected socioeconomic and demographic variables using Wave 21 data, with all estimates weighted using HILDA population weights. At the national level, Fig. 3 shows the weighted annual prevalence of meal skipping due to financial constraint between 2001 and 2021, based on ∼13 000–15 000 SCQ respondents per wave (Supplementary Table S3). Prevalence fluctuated between 3.0% and 4.6% across the 21-year period, with the highest weighted estimate in 2001 (4.6%) and the lowest in 2006 (3.0%).

The COVID years of 2020 and 2021 (highlighted in Fig. 3) showed prevalence of 3.2% and 3.4% respectively, which are below the 2019 peak of 4.3%, a pattern consistent with the temporary expansion of income support through the JobKeeper and JobSeeker programmes. A sensitivity analysis excluding the COVID waves (2020–2021) confirmed that the income gradient in meal skipping and the Engel curve relationship were qualitatively unchanged (Supplementary Table S1). Among welfare recipients specifically, weighted meal-skipping prevalence fell from 7.7% in 2019 (pre-COVID) to 5.8% in 2020% and 6.2% in 2021. Among nonwelfare recipients, prevalence fell from 2.4% in 2019 to 1.6% in 2020. These patterns are consistent with the temporary income support provided through JobKeeper (a COVID-era wage subsidy, March 2020–March 2021) and the temporarily doubled JobSeeker (unemployment) payment, which may have suppressed meal skipping among the most vulnerable households during the pandemic period.

Results were also robust to trimming at the tails of the income distribution (Supplementary Table S2). Excluding households with equivalized income below $5000 per year or below the first percentile ($6527), and capping food expenditure share at the 99th percentile, did not substantively alter the income coefficient in either the logit or Engel curve specifications. The finding that some households spend more than half their equivalized income on food survived all trimming approaches (13 681 households after the $5000 floor, compared with 15 184 in the untrimmed sample).

**Figure 4:**
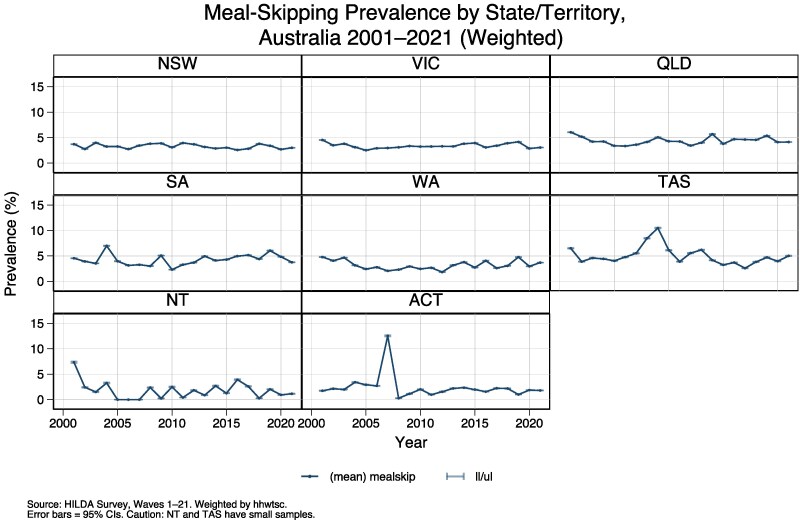
Annual prevalence of meal skipping due to financial constraint by state/territory (Australia, 2001–2021).

The panel in Fig. 4 disaggregates the prevalence of meal skipping by Australian states and territories from 2001 to 2021. The variation across jurisdictions suggests that food insecurity is not uniformly distributed across Australia, with certain states/territories, such as the Northern Territory (NT) and Tasmania (TAS), exhibiting higher fluctuations and, in some cases, a higher overall prevalence. Other states, such as New South Wales (NSW) and Victoria (VIC), show relatively stable trends, though still with observable periods of increase. The differences across states and territories may reflect regional economic disparities, differences in cost-of-living pressures, and access to social support mechanisms.

A descriptive analysis of repeat reporting is provided in Supplementary Table S4. Among 5136 respondents in the balanced panel (all 21 waves), 85.0% never reported meal skipping, 5.8% reported it in exactly one wave, 9.1% reported it in two or more waves but not every wave, and only 2 individuals (0.04%) reported it in every wave. This indicates that meal skipping is episodic for most who experience it, but recurrent for a meaningful minority.

The HILDA survey includes a question whether respondents ‘asked welfare/community organizations for help’ (fiprbwo, SCQ C2 g). Among all respondents who reported meal skipping, 35.4% also reported asking welfare or community organizations for help (weighted), compared with 2.6% of nonskippers. Among bottom-quintile respondents who sought help from welfare organizations, 58.4% did not report meal skipping, which is consistent with the hypothesis that accessing food relief may buffer against the most severe food insecurity outcomes (Supplementary Table S5). However, HILDA’s fiprbwo variable is broader than just foodbank use, so this relationship should be interpreted cautiously.

**Figure 5:**
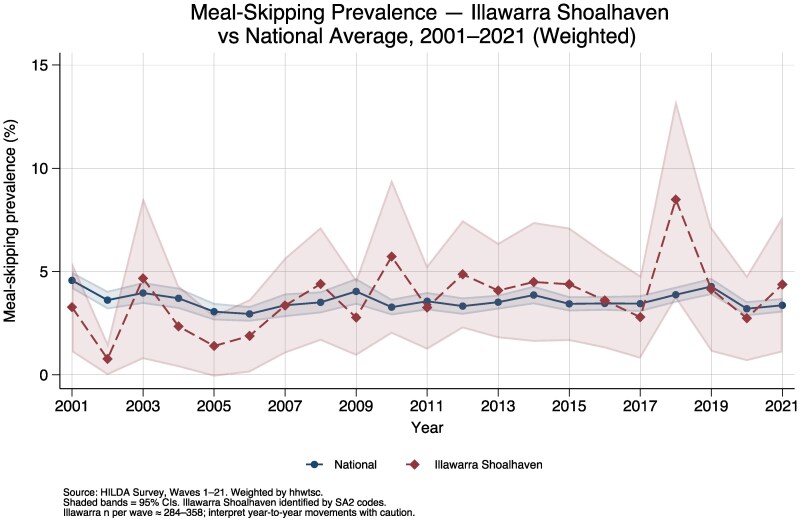
Meal-skipping prevalence—Illawarra Shoalhaven vs National Average, 2001–2021.

We formally tested whether meal-skipping prevalence in the Illawarra Shoalhaven region differed from the national average using a weighted logistic regression with an Illawarra indicator and Illawarra-by-wave interaction terms. The Illawarra main effect was not statistically significant (coefficient: −0.35, SE: 0.35, *F* = 1.01, *P* = 0.315), and pooled weighted prevalence was similar: 3.7% in the Illawarra Shoalhaven (95% CI: 2.4%–4.9%) versus 3.6% nationally (95% CI: 3.4%–3.8%). The joint test of the Illawarra dummy and all 21 wave interactions was borderline significant (*F* = 1.82, *P* = 0.012), but this likely reflects noisy year-specific deviations rather than a systematic regional effect, given that no individual interaction term was consistently significant and the Illawarra subsample ranged from only 284 to 358 individuals per wave. Figure 5 illustrates the year-by-year comparison, with substantially wider confidence intervals for the Illawarra series. An attrition analysis showed that Illawarra respondents who left the panel before Wave 21 had significantly lower baseline income ($34 899 vs $41 486 for stayers, *P* < 0.001) and were more likely to be welfare recipients (45.6% vs 31.8%, *P* < 0.001), suggesting that the Illawarra prevalence estimate may be biased downward by selective attrition of more disadvantaged respondents, making the null finding conservative. Among the 694 unique respondents who ever appeared in the Illawarra Shoalhaven subsample with a valid meal-skip response, the mean number of waves contributed was 8, with a median of 6.

To assess the extent of within-household measurement heterogeneity, we examined multi-adult households where two or more members completed the SCQ meal-skipping item. In 4.55% of such households, at least one member reported skipping meals while another did not, confirming that intra-household variation in food insecurity reporting exists but is relatively uncommon. This finding supports the use of household-level income as a predictor of individual-level meal skipping while acknowledging a modest degree of measurement noise at the household level.

## Discussion

This study examined the association between household income and severe food insecurity in Australia and assessed how income-related patterns vary across geographic scales using longitudinal data from the HILDA survey. Using meal skipping due to financial constraint as an indicator of severe food insecurity, alongside household food expenditure share as a measure of food-related economic pressure, the analysis provides evidence of a strong and persistent income gradient in food insecurity at the national level. A formal test of whether meal-skipping prevalence in the Illawarra Shoalhaven region differed from the national average returned no statistically significant difference, suggesting that the income gradient generalizes across geographic contexts rather than reflecting localized disadvantage. Together, the findings help clarify how food insecurity manifests in high-income settings and why national prevalence estimates alone provide a potentially incomplete account of household vulnerability.

Our analysis demonstrates a clear relationship between household income and the likelihood of skipping meals due to financial constraint, with severe food insecurity concentrated most heavily among households at the lower end of the income distribution. This strong income gradient is consistent with prior Australian and international evidence identifying income adequacy as the strongest observed correlate of food insecurity in high-income countries (Gatton and Gallegos 2023), particularly among households reliant on welfare payments or exposed to financial shocks (Temple *et al*. 2019). The strength of this association is consistent with food insecurity in Australia being primarily associated with structural economic factors rather than individual behaviours. In a highly concentrated supermarket sector, where two retailers control the majority of the grocery market (Coles and Woolworths together account for ∼65% of grocery sales), households have limited capacity to avoid or offset these price increases. For low-income households, this likely reflects sustained trade-offs between food and other essentials such as housing, utilities, transport, and healthcare (Seivwright *et al*. 2024). In this context, meal skipping represents the extreme end of a broader continuum of food insecurity, occurring only after less visible coping strategies such as reducing diet quality, food variety, or portion sizes have already been exhausted (Lindberg *et al*. 2022).

The accompanying analysis of household food expenditure share provides complementary evidence of underlying economic pressure that precedes observable behavioural indicators of severe food insecurity. Spending more than 30% of household income on food is widely considered indicative of severe financial strain (Burns and Friel 2007); yet, the results show that this threshold is exceeded not only among very low-income households but also among some households with moderate incomes. This pattern aligns with broader evidence that rising food prices, rather than absolute food availability, are increasingly central to food insecurity in high-income settings (Headey *et al*. 2024). This helps explain why food stress and food relief use are increasingly reported among households that do not fit traditional poverty definitions. High food expenditure as a proportion of total income constrains a household’s capacity to absorb income shocks or price increases, making food insecurity more likely during periods of inflation, housing stress, or employment instability (Wood *et al*. 2023). These income pressures have been exacerbated by sustained increases in the price of staple foods, with national data showing a 15.2% rise in the cost of a basic grocery basket between 2021 and 2023, including much larger increases for core items such as bread, milk, cheese, and eggs (Nurdi 2024). This financial pressure is strongly associated with poorer diet quality, as households prioritize energy-dense, low-cost foods over fresh and nutritious options (Kent *et al*. 2024), contributing to well-documented links between food insecurity, chronic disease, and poor mental health outcomes (Kent *et al*. 2025). These findings reinforce that food insecurity is both a nutritional and health equity issue (Zorbas *et al*. 2021) rooted in income adequacy rather than food availability alone. Under the groceries-only definition, 15% of Wave 21 households exceeded the 30% threshold, compared with 27% under the total food definition. This indicates that for roughly half of the households exceeding the 30% threshold, expenditure on meals out contributes meaningfully to their total food expenditure share. However, the groceries-only specification narrows but does not eliminate the headline finding: a substantial proportion of low-income households still devote a large share of their income to food even when meals out are excluded, and the downward-sloping Engel curve relationship is robust to both definitions.

To better characterize the nature of repeated food insecurity, we examined individual-level reporting patterns across the balanced panel of 5136 respondents observed in all 21 waves. Among these respondents, 85.0% never reported meal skipping; 5.8% reported it in exactly one wave; 9.1% reported it in two or more waves but not in every wave; and only two individuals (0.04%) reported meal skipping in every observed wave. This pattern indicates that meal skipping at this severity threshold is episodic for most who experience it, consistent with transient responses to income shocks, job loss, or unexpected expenses, but recurrent for a meaningful minority. The 9.1% of balanced-panel respondents who reported meal skipping in two or more waves represent a group for whom food insecurity is not a one-off event, even if it does not occur in every year. The data support a pattern of recurring but intermittent food insecurity rather than chronic, unbroken hardship. Nevertheless, the population-level persistence of a 3%–5% annual prevalence rate across two decades confirms that the structural correlates of severe food insecurity, principally income inadequacy relative to living costs, remain largely unchanged, and that food charity and emergency relief, while important for alleviating immediate hunger (Lindberg *et al*. 2015), do not address these underlying factors (McKay and Lindberg 2019). Without coordinated policy action across income support, housing affordability, and social protection systems, severe food insecurity will continue to recur at the population level even as individual households cycle in and out of acute food stress.

Importantly, this analysis demonstrates that severe food insecurity is not uniformly distributed across Australia. By disaggregating prevalence by state and territory, we show that there is geographic variation in food insecurity, with smaller jurisdictions such as the Northern Territory and Tasmania exhibiting greater volatility and, in some periods, higher levels of meal skipping than larger states such as New South Wales and Victoria. This is mirrored in national research into food insecurity by charitable agencies (Foodbank Australia 2023). While estimates for smaller jurisdictions are subject to greater uncertainty due to sample size limitations, the repeated emergence of state-level differences across years suggests that these patterns are unlikely to reflect random variation alone. Rather, they point to the influence of structural, place-based factors including high housing costs, labour market insecurity, geographic isolation, access to services, and food retail environments that interact with household income to shape food insecurity risk. Given state-level responsibility for housing, health services, and elements of employment policy, our findings underscore the importance of place-sensitive, policy-aligned responses, alongside national income adequacy reforms, to meaningfully reduce severe food insecurity in Australia.

When the Illawarra Shoalhaven region is compared with the national average, meal-skipping prevalence does not differ significantly, suggesting that the income gradient documented above generalizes across regions rather than reflecting localized disadvantage. This conclusion should be read alongside the attrition pattern in our sample: lower-income respondents were significantly more likely to leave the panel, which would tend to attenuate any true regional gap and makes our null finding conservative.

Importantly, this finding does not imply that food insecurity is not a concern in the Illawarra Shoalhaven—local surveys using broader measures (e.g. the 18-item HFSSM) consistently report higher prevalence (Kent *et al*. 2025), and community demand for food relief continues to grow (Cartwright 2025). Rather, it suggests that when measured by the single severe indicator of meal skipping available in HILDA, the Illawarra Shoalhaven does not differ detectably from the national pattern, and that meal skipping in Australia is best understood as a consequence of position in the national income distribution, with regional context playing a secondary role. The contrast between our null HILDA finding and higher prevalence estimates from dedicated regional surveys likely reflects the broader scope of multi-item food insecurity measures, differences in sampling, and the substantially larger samples available in purpose-designed local studies.

The COVID-19 pandemic period offers a natural policy experiment relevant to the income-gradient findings. Among welfare recipients, weighted meal-skipping prevalence fell from 7.7% in 2019 to 5.8% in 2020% and 6.2% in 2021; among nonwelfare recipients, it fell from 2.4% to 1.6% in 2020. This pattern is consistent with the temporary expansion of income support through the doubled JobSeeker payment and the introduction of JobKeeper, which temporarily increased disposable income for the most vulnerable households. The subsequent partial rebound in 2021, as these supports were withdrawn, further reinforces the centrality of income adequacy. These findings add to growing Australian evidence that targeted income transfers can meaningfully reduce severe food insecurity (Botha and Payne 2022) and suggest that the structural persistence of meal skipping documented in our 21-year series is not inevitable but responsive to policy intervention.

Among all respondents who reported meal skipping, over a third also reported asking welfare or community organizations for help (weighted), compared with <3% of those who did not skip meals. Among bottom-quintile respondents who sought help from welfare organizations, the majority (58.4%) did not report meal skipping, which is consistent with the hypothesis that accessing food relief or community support may buffer against the most severe food insecurity outcomes. However, HILDA does not directly measure foodbank use, and the variable used captures a broader range of welfare-seeking behaviour, so this relationship should be interpreted cautiously. The finding that almost two-thirds of meal skippers did not seek welfare support suggests that unmet need remains considerable and that many food-insecure individuals do not access available services.

Our weighted Wave 21 estimate of 3.4% for meal skipping is substantially lower than the ABS estimate of 8.5% moderate or severe food insecurity (ABS 2025), a difference that is expected and informative. The ABS figure is based on the eight-item Food Insecurity Experience Scale (FIES), which captures a broader range of food insecurity experiences including worry about running out of food, inability to eat healthy and nutritious food, and eating less than one should. Meal skipping is one of the more severe items within such scales, and our estimate should therefore be understood as capturing the severe tail of food insecurity. Reference period and sampling differences may also contribute. The gap between the two estimates contextualizes our prevalence as conservative and underscores that a much larger share of the Australian population experiences food insecurity at lower severity thresholds than our single-item measure captures.

Taken together, these findings reinforce the centrality of income gradients in explaining severe food insecurity at the national level. The absence of a statistically significant regional difference in meal-skipping prevalence between the Illawarra Shoalhaven and the national average suggests that position in the income distribution is a stronger predictor of severe food insecurity than regional context *per se*. However, national data using single-item measures capture only the severe tail of food insecurity, and regional surveys using multi-item instruments consistently identify broader and more prevalent food-related vulnerability. National surveillance and local evidence thus serve complementary functions: the former identifies structural patterns and income gradients; the latter captures the fuller spectrum of food insecurity experience and community-level service demand. For health promotion practitioners and policymakers, integrating surveillance with regional analysis enables more targeted, equity-oriented planning and resource allocation. A robust national food security monitoring system should therefore integrate income, expenditure, and food insecurity measures, while enabling regional disaggregation and linkage with local data sources (Godrich *et al*. 2021). Critically, food security policy must centre income adequacy as a core pillar. Without addressing the affordability of basic living costs through income support, housing policy, and employment conditions, place-based food initiatives will remain necessary but insufficient (Godrich *et al*. 2021). Food insecurity is ultimately experienced locally, but it is produced structurally, and effective policy must operate across federal, state and local-level scales.

### Limitations and future research

Drawing on the framework outlined by Hernán (2018), we treat this study as a test of a causal model in which insufficient income contributes to severe food insecurity, even though the data we use are observational and predominantly cross-sectional. The analysis cannot establish a causal pathway on its own; rather, it asks whether the associations we observe in 21 waves of nationally representative data are consistent with that model and how strong the implied gradient is. We therefore present associations as evidence consistent with a causal process while remaining explicit that the strength of the inferences about a true causal effect is limited by the study design, by the potential for reverse causation, and by unobserved confounding.

This study has several limitations that should be considered when interpreting the findings. First, although HILDA provides a longitudinal panel structure, our primary analyses treat the data as repeated cross-sections. As noted above, the observational nature of the data and the predominantly cross-sectional design limit the strength of the causal inferences that can be drawn. The estimates may be affected by reverse causation (e.g. food insecurity feeding back into employment and income) and by residual confounding from unobserved factors. Future research should exploit the longitudinal structure of HILDA to examine transitions into and out of food insecurity, duration dependence, and the role of income shocks versus chronic low income.

Second, our primary indicator—meal skipping due to financial constraint—captures only the severe tail of food insecurity. Multi-item scales such as the eight-item FIES used by the ABS identify a broader range of food insecurity experiences; our estimate of 3.4% (Wave 21, weighted) compared with the ABS figure of 8.5% illustrates the substantial difference in scope. Milder but consequential forms of food stress—reduced diet quality, smaller portions, reliance on energy-dense but nutrient-poor foods—are not captured by our measure.

Third, HILDA does not consistently capture food-charity or foodbank use. The closest proxy (fiprbwo): asked welfare or community organizations for help is broader than foodbank use and is not a direct measure of food relief access. This limits our ability to test whether informal food sources mediate the income-meal-skipping relationship.

Fourth, within-household measurement heterogeneity is nontrivial: 4.55% of multi-adult households contain members who disagree on the meal-skipping item, indicating that household-level income does not perfectly predict individual-level food insecurity experience. Our clustering strategy (on persistent person identifier for pooled models) addresses repeated observations but does not resolve this within-household noise.

Fifth, the Illawarra Shoalhaven regional analysis is affected by selective attrition: lower-income respondents were significantly more likely to leave the panel than higher-income respondents. Because attrition is concentrated among the group most at risk of food insecurity, any true regional gap in meal-skipping prevalence would tend to be attenuated, and the absence of a significant regional difference in our HILDA-based comparison should therefore be interpreted as conservative.

Sixth, self-reported financial stress items are subject to recall bias and social desirability effects. Respondents may under-report meal skipping due to stigma, and a person reporting meal skipping in only one wave may be experiencing chronic food insecurity that they disclosed only once. The repeat-reporting analysis therefore provides a lower bound on the true prevalence of recurring food insecurity.

Despite these limitations, the study provides the first weighted, population-representative analysis of severe food insecurity across 21 waves of HILDA, with formal regression models, sensitivity analyses, and a structured regional comparison, strengthening the evidence base for income-focused policy responses to food insecurity in Australia.

## Conclusion

Using two decades of nationally representative, population-weighted household data, this study documents a strong and persistent association between income and severe food insecurity in Australia. Households at the lower end of the income distribution face substantially higher probabilities of meal skipping and devote a disproportionate share of their income to food. For most individuals who experience it, meal skipping is episodic rather than chronic, though a meaningful minority report it repeatedly across waves. A formal comparison of the Illawarra Shoalhaven region with the national average found no statistically significant difference, suggesting that the income gradient generalizes across geographic contexts and that meal skipping is best understood as a consequence of position in the national income distribution. The temporary reduction in meal skipping among welfare recipients during the COVID-19 income support expansion further reinforces income adequacy as the central policy lever. Reducing severe food insecurity in Australia will require coordinated, multi-level action centred on strengthening income support and social protection, alongside place-based responses addressing local cost pressures, food environments, and access to essential services.

## Supplementary Material

daag079_Supplementary_Data

## Data Availability

The data used in this study are from the confidentialized unit record file of the Household, Income and Labour Dynamics in Australia (HILDA) Survey, Release 21, managed by the Melbourne Institute of Applied Economic and Social Research. Under the terms of the licence agreement, the authors cannot redistribute these data. Researchers wishing to access HILDA data should apply through the Australian Data Archive (https://dataverse.ada.edu.au) or the Melbourne Institute. Analysis code is available from the corresponding author on reasonable request.
