# Supplementary material for: Income, food expenditure shares, and severe food insecurity in Australia across 21 waves of HILDA
Source: Health Promot Int. 2026 Jun 4;41(3):daag079. doi: 10.1093/heapro/daag079 (PMC13234612; doi:10.1093/heapro/daag079)
Supplement: daag079_Supplementary_Data [file daag079_supplementary_data.zip › tab_s1_covid_sensitivity_ACCEPTED.docx]

Table S1: COVID Sensitivity — Main Models With and Without 2020–2021

|  | (1) | (2) | (3) | (4) |
| --- | --- | --- | --- | --- |
|  | Logit (all) | Logit (excl. COVID) | Engel (all) | Engel (excl. COVID) |
| main |  |  |  |  |
| Equivalised household disposable income (annual) | -0.000^***^ | -0.000^***^ |  |  |
|  | (0.000) | (0.000) |  |  |
|  |  |  |  |  |
| DV: Age last birthday at June 30 2001 | 0.095^***^ | 0.090^***^ |  |  |
|  | (0.010) | (0.010) |  |  |
|  |  |  |  |  |
| Age squared | -0.002^***^ | -0.001^***^ |  |  |
|  | (0.000) | (0.000) |  |  |
|  |  |  |  |  |
|  |  |  |  |  |
| Female (1=Yes)=1 | -0.133^**^ | -0.145^***^ |  |  |
|  | (0.055) | (0.056) |  |  |
|  |  |  |  |  |
| DV: Number of persons aged 15+ years at June 30 2001 | -0.250^***^ | -0.249^***^ | 2.021^***^ | 2.234^***^ |
|  | (0.030) | (0.031) | (0.259) | (0.272) |
|  |  |  |  |  |
| DV: Number of dependent children aged 0-4 (includes partner's children) | -0.322^***^ | -0.322^***^ | -0.576^***^ | -0.462^**^ |
|  | (0.044) | (0.046) | (0.197) | (0.213) |
|  |  |  |  |  |
| DV: Number of dependent children aged 5-9 (includes partner's children) | -0.297^***^ | -0.292^***^ | 0.834^***^ | 1.005^***^ |
|  | (0.049) | (0.052) | (0.200) | (0.217) |
|  |  |  |  |  |
| DV: Number of dependent children aged 10-14 (includes partner's children) | -0.216^***^ | -0.215^***^ | 1.879^***^ | 2.064^***^ |
|  | (0.049) | (0.052) | (0.202) | (0.218) |
|  |  |  |  |  |
| DV: Number of dependent children aged 15-24 (includes partner's children) | 0.090 | 0.090 | 0.908^***^ | 0.977^***^ |
|  | (0.061) | (0.065) | (0.157) | (0.165) |
|  |  |  |  |  |
|  |  |  |  |  |
| Indigenous (ATSI) (1=Yes)=1 | 0.060 | 0.061 |  |  |
|  | (0.105) | (0.108) |  |  |
|  |  |  |  |  |
|  |  |  |  |  |
| Lives in major city (1=Yes)=1 | -0.032 | -0.045 | 0.050 | 0.044 |
|  | (0.057) | (0.059) | (0.122) | (0.129) |
|  |  |  |  |  |
|  |  |  |  |  |
| [2] Unemployed | 0.727^***^ | 0.739^***^ |  |  |
|  | (0.062) | (0.065) |  |  |
|  |  |  |  |  |
| [3] Not in the labour force | 0.332^***^ | 0.309^***^ |  |  |
|  | (0.055) | (0.058) |  |  |
|  |  |  |  |  |
|  |  |  |  |  |
| Rents current dwelling (1=Yes)=1 | 0.762^***^ | 0.787^***^ |  |  |
|  | (0.058) | (0.060) |  |  |
|  |  |  |  |  |
|  |  |  |  |  |
| Receives welfare/transfer income (1=Yes)=1 | 0.719^***^ | 0.711^***^ |  |  |
|  | (0.051) | (0.054) |  |  |
|  |  |  |  |  |
|  |  |  |  |  |
| Self-assessed health (1=poor ... 5=excellent)=2 | -0.479^***^ | -0.462^***^ |  |  |
|  | (0.088) | (0.093) |  |  |
|  |  |  |  |  |
| Self-assessed health (1=poor ... 5=excellent)=3 | -1.064^***^ | -1.061^***^ |  |  |
|  | (0.093) | (0.098) |  |  |
|  |  |  |  |  |
| Self-assessed health (1=poor ... 5=excellent)=4 | -1.613^***^ | -1.593^***^ |  |  |
|  | (0.101) | (0.106) |  |  |
|  |  |  |  |  |
| Self-assessed health (1=poor ... 5=excellent)=5 | -1.892^***^ | -1.886^***^ |  |  |
|  | (0.117) | (0.125) |  |  |
|  |  |  |  |  |
| Log equivalised household income |  |  | -16.566^***^ | -16.782^***^ |
|  |  |  | (0.146) | (0.153) |
|  |  |  |  |  |
| HF Number of in-scope persons in household |  |  | 2.633^***^ | 2.516^***^ |
|  |  |  | (0.188) | (0.203) |
|  |  |  |  |  |
| Constant | -2.584^***^ | -2.491^***^ | 180.600^***^ | 182.585^***^ |
|  | (0.243) | (0.254) | (1.380) | (1.448) |
| Observations | 204,930 | 181,400 | 232,985 | 200,811 |
| Pseudo R-sq | 0.192 | 0.190 |  |  |
| R-squared |  |  | 0.504 | 0.503 |
| N_clusters | 23,855 | 22,829 | 31,666 | 30,472 |

Logit weighted by hhwtsc; Engel weighted by hhwtrp. SEs clustered on xwaveid. COVID waves = 20 (2020) and 21 (2021).

^*^ *p* < 0.10, ^**^ *p* < 0.05, ^***^ *p* < 0.01
