# Supplementary material for: Income, food expenditure shares, and severe food insecurity in Australia across 21 waves of HILDA
Source: Health Promot Int. 2026 Jun 4;41(3):daag079. doi: 10.1093/heapro/daag079 (PMC13234612; doi:10.1093/heapro/daag079)
Supplement: daag079_Supplementary_Data [file daag079_supplementary_data.zip › tab_s2_trimming_ACCEPTED.docx]

Table S2: Sensitivity to Income/Expenditure Trimming

|  | (1) | (2) | (3) | (4) |
| --- | --- | --- | --- | --- |
|  | Logit (>=$5k) | Logit (>=p1) | Engel (>=$5k) | Engel (>=p1) |
| main |  |  |  |  |
| Equivalised household disposable income (annual) | -0.000^***^ | -0.000^***^ |  |  |
|  | (0.000) | (0.000) |  |  |
|  |  |  |  |  |
| DV: Age last birthday at June 30 2001 | 0.110^***^ | 0.110^***^ |  |  |
|  | (0.011) | (0.011) |  |  |
|  |  |  |  |  |
| Age squared | -0.002^***^ | -0.002^***^ |  |  |
|  | (0.000) | (0.000) |  |  |
|  |  |  |  |  |
|  |  |  |  |  |
| Female (1=Yes)=1 | -0.112^*^ | -0.112^*^ |  |  |
|  | (0.057) | (0.057) |  |  |
|  |  |  |  |  |
| DV: Number of persons aged 15+ years at June 30 2001 | -0.232^***^ | -0.231^***^ | 2.018^***^ | 2.004^***^ |
|  | (0.033) | (0.033) | (0.260) | (0.260) |
|  |  |  |  |  |
| DV: Number of dependent children aged 0-4 (includes partner's children) | -0.325^***^ | -0.325^***^ | -0.617^***^ | -0.625^***^ |
|  | (0.047) | (0.047) | (0.200) | (0.200) |
|  |  |  |  |  |
| DV: Number of dependent children aged 5-9 (includes partner's children) | -0.332^***^ | -0.331^***^ | 0.858^***^ | 0.851^***^ |
|  | (0.051) | (0.051) | (0.205) | (0.205) |
|  |  |  |  |  |
| DV: Number of dependent children aged 10-14 (includes partner's children) | -0.237^***^ | -0.235^***^ | 1.838^***^ | 1.840^***^ |
|  | (0.052) | (0.052) | (0.207) | (0.207) |
|  |  |  |  |  |
| DV: Number of dependent children aged 15-24 (includes partner's children) | 0.043 | 0.046 | 0.937^***^ | 0.935^***^ |
|  | (0.071) | (0.071) | (0.159) | (0.159) |
|  |  |  |  |  |
|  |  |  |  |  |
| Indigenous (ATSI) (1=Yes)=1 | 0.114 | 0.110 |  |  |
|  | (0.114) | (0.115) |  |  |
|  |  |  |  |  |
|  |  |  |  |  |
| Lives in major city (1=Yes)=1 | -0.039 | -0.040 | 0.112 | 0.096 |
|  | (0.060) | (0.060) | (0.125) | (0.125) |
|  |  |  |  |  |
|  |  |  |  |  |
| [2] Unemployed | 0.709^***^ | 0.705^***^ |  |  |
|  | (0.070) | (0.070) |  |  |
|  |  |  |  |  |
| [3] Not in the labour force | 0.362^***^ | 0.361^***^ |  |  |
|  | (0.060) | (0.060) |  |  |
|  |  |  |  |  |
|  |  |  |  |  |
| Rents current dwelling (1=Yes)=1 | 0.682^***^ | 0.683^***^ |  |  |
|  | (0.062) | (0.063) |  |  |
|  |  |  |  |  |
|  |  |  |  |  |
| Receives welfare/transfer income (1=Yes)=1 | 0.719^***^ | 0.719^***^ |  |  |
|  | (0.057) | (0.057) |  |  |
|  |  |  |  |  |
|  |  |  |  |  |
| Self-assessed health (1=poor ... 5=excellent)=2 | -0.532^***^ | -0.539^***^ |  |  |
|  | (0.095) | (0.095) |  |  |
|  |  |  |  |  |
| Self-assessed health (1=poor ... 5=excellent)=3 | -1.082^***^ | -1.091^***^ |  |  |
|  | (0.098) | (0.098) |  |  |
|  |  |  |  |  |
| Self-assessed health (1=poor ... 5=excellent)=4 | -1.621^***^ | -1.628^***^ |  |  |
|  | (0.106) | (0.106) |  |  |
|  |  |  |  |  |
| Self-assessed health (1=poor ... 5=excellent)=5 | -1.928^***^ | -1.945^***^ |  |  |
|  | (0.128) | (0.128) |  |  |
|  |  |  |  |  |
| Log equivalised household income |  |  | -16.901^***^ | -16.761^***^ |
|  |  |  | (0.151) | (0.153) |
|  |  |  |  |  |
| HF Number of in-scope persons in household |  |  | 2.681^***^ | 2.695^***^ |
|  |  |  | (0.191) | (0.192) |
|  |  |  |  |  |
| Constant | -2.818^***^ | -2.839^***^ | 184.028^***^ | 182.528^***^ |
|  | (0.267) | (0.269) | (1.451) | (1.469) |
| Observations | 152,009 | 151,761 | 232,960 | 232,483 |
| Pseudo R-sq | 0.193 | 0.193 |  |  |
| R-squared |  |  | 0.503 | 0.500 |
| N_clusters | 22,167 | 22,150 | 31,664 | 31,640 |

Logit: hhwtsc; Engel: hhwtrp. SEs clustered on xwaveid. All samples also trim food expenditure share at 99th percentile. 1st percentile of equiv_income = $6526.666666666667.

^*^ *p* < 0.10, ^**^ *p* < 0.05, ^***^ *p* < 0.01
