# Supplementary material for: Income, food expenditure shares, and severe food insecurity in Australia across 21 waves of HILDA
Source: Health Promot Int. 2026 Jun 4;41(3):daag079. doi: 10.1093/heapro/daag079 (PMC13234612; doi:10.1093/heapro/daag079)
Supplement: daag079_Supplementary_Data [file daag079_supplementary_data.zip › tab_s3_sample_sizes.docx]

Table S3: Sample Sizes by Wave

| **Wave** | **Total PQ respondents 15+** | **SCQ respondents** | **Valid fiprbwm** | **Analysis sample** |
| --- | --- | --- | --- | --- |
| 2001 | 13665 | 12764 | 12556 | 12506 |
| 2002 | 12711 | 11831 | 11688 | 11634 |
| 2003 | 12410 | 11470 | 11304 | 11250 |
| 2004 | 12072 | 11102 | 10892 | 10827 |
| 2005 | 12421 | 11170 | 11068 | 11009 |
| 2006 | 12523 | 11351 | 11157 | 11093 |
| 2007 | 12468 | 11109 | 10906 | 10853 |
| 2008 | 12425 | 10896 | 10060 | 9999 |
| 2009 | 12948 | 11274 | 11148 | 11080 |
| 2010 | 13174 | 11762 | 11106 | 11049 |
| 2011 | 17172 | 15016 | 14908 | 14812 |
| 2012 | 17004 | 15017 | 14254 | 14147 |
| 2013 | 17019 | 14975 | 14884 | 14745 |
| 2014 | 16991 | 15172 | 14725 | 14581 |
| 2015 | 17083 | 15099 | 15073 | 14919 |
| 2016 | 17191 | 15836 | 15710 | 15527 |
| 2017 | 17091 | 15752 | 14938 | 14767 |
| 2018 | 16924 | 15465 | 15044 | 14870 |
| 2019 | 16945 | 15639 | 15537 | 15328 |
| 2020 | 16580 | 15257 | 15164 | 14948 |
| 2021 | 16049 | 14874 | 14629 | 14420 |
| **Total** | **312866** | **282831** | **276751** | **274364** |
