# Supplementary material for: Income, food expenditure shares, and severe food insecurity in Australia across 21 waves of HILDA
Source: Health Promot Int. 2026 Jun 4;41(3):daag079. doi: 10.1093/heapro/daag079 (PMC13234612; doi:10.1093/heapro/daag079)
Supplement: daag079_Supplementary_Data [file daag079_supplementary_data.zip › tab_s4_repeat_reporting.docx]

Table S4: Repeat Reporting of Meal-Skipping (Balanced Panel, 21 Waves)

|  | (1) |
| --- | --- |
|  | ms_category |
|  | b/pct |
| Never reported | 4177 |
|  | 85.0 |
| Reported in 1 wave | 287 |
|  | 5.8 |
| Reported in 2+ waves (not all) | 449 |
|  | 9.1 |
| Reported in every wave | 2 |
|  | 0.0 |
| Total | 4915 |
|  | 100.0 |
| *N* | 4915 |

Balanced panel: respondents present in all 21 waves. Categories based on count of waves with mealskip = 1.
