# Supplementary material for: Income, food expenditure shares, and severe food insecurity in Australia across 21 waves of HILDA
Source: Health Promot Int. 2026 Jun 4;41(3):daag079. doi: 10.1093/heapro/daag079 (PMC13234612; doi:10.1093/heapro/daag079)
Supplement: daag079_Supplementary_Data [file daag079_supplementary_data.zip › tab_s5_foodbank_proxy.docx]

Table S5: Meal-Skipping × Asked Welfare/Community Organisations

|  | (1) |  |  |
| --- | --- | --- | --- |
|  |  |  |  |
|  | 0 | 1 | Total |
|  | b | b | b |
| No | 245849 | 6545 | 252394 |
| Yes | 6425 | 3666 | 10091 |
| Total | 252274 | 10211 | 262485 |
| *N* | 262485 |  |  |

Unweighted counts. fiprbwo = SCQ C2g (asked welfare organisations for help).
