# Supplementary material for: Income, food expenditure shares, and severe food insecurity in Australia across 21 waves of HILDA
Source: Health Promot Int. 2026 Jun 4;41(3):daag079. doi: 10.1093/heapro/daag079 (PMC13234612; doi:10.1093/heapro/daag079)
Supplement: daag079_Supplementary_Data [file daag079_supplementary_data.zip › tab_s6_logit_mealskip_v2_ACCEPTED.docx]

Table S6: Logit Model — Probability of Skipping a Meal

|  | (1) | (2) | (3) | (4) |
| --- | --- | --- | --- | --- |
|  | Bivariate | Demographics | Full | COVID sensitivity |
| Skipped a meal due to money shortage (SCQ C2d) |  |  |  |  |
| Equivalised household disposable income (annual) | -0.000^***^ | -0.000^***^ | -0.000^***^ | -0.000^***^ |
|  | (0.000) | (0.000) | (0.000) | (0.000) |
|  |  |  |  |  |
| DV: Age last birthday at June 30 2001 |  | 0.112^***^ | 0.095^***^ | 0.095^***^ |
|  |  | (0.010) | (0.010) | (0.010) |
|  |  |  |  |  |
| Age squared |  | -0.002^***^ | -0.002^***^ | -0.002^***^ |
|  |  | (0.000) | (0.000) | (0.000) |
|  |  |  |  |  |
|  |  |  |  |  |
| Female (1=Yes)=1 |  | -0.053 | -0.133^**^ | -0.133^**^ |
|  |  | (0.053) | (0.055) | (0.055) |
|  |  |  |  |  |
| DV: Number of persons aged 15+ years at June 30 2001 |  | -0.257^***^ | -0.250^***^ | -0.250^***^ |
|  |  | (0.027) | (0.030) | (0.030) |
|  |  |  |  |  |
| DV: Number of dependent children aged 0-4 (includes partner's children) |  | -0.413^***^ | -0.322^***^ | -0.322^***^ |
|  |  | (0.041) | (0.044) | (0.044) |
|  |  |  |  |  |
| DV: Number of dependent children aged 5-9 (includes partner's children) |  | -0.374^***^ | -0.297^***^ | -0.297^***^ |
|  |  | (0.046) | (0.049) | (0.049) |
|  |  |  |  |  |
| DV: Number of dependent children aged 10-14 (includes partner's children) |  | -0.334^***^ | -0.216^***^ | -0.217^***^ |
|  |  | (0.047) | (0.049) | (0.049) |
|  |  |  |  |  |
| DV: Number of dependent children aged 15-24 (includes partner's children) |  | -0.101 | 0.090 | 0.090 |
|  |  | (0.062) | (0.061) | (0.061) |
|  |  |  |  |  |
|  |  |  |  |  |
| Indigenous (ATSI) (1=Yes)=1 |  | 0.523^***^ | 0.060 | 0.060 |
|  |  | (0.099) | (0.105) | (0.105) |
|  |  |  |  |  |
|  |  |  |  |  |
| VIC |  | -0.025 | 0.020 | 0.020 |
|  |  | (0.075) | (0.076) | (0.076) |
|  |  |  |  |  |
| QLD |  | 0.126^*^ | 0.083 | 0.083 |
|  |  | (0.070) | (0.073) | (0.073) |
|  |  |  |  |  |
| SA |  | 0.032 | -0.046 | -0.046 |
|  |  | (0.097) | (0.102) | (0.102) |
|  |  |  |  |  |
| WA |  | 0.016 | 0.050 | 0.050 |
|  |  | (0.105) | (0.108) | (0.108) |
|  |  |  |  |  |
| TAS |  | 0.040 | -0.047 | -0.047 |
|  |  | (0.139) | (0.135) | (0.135) |
|  |  |  |  |  |
| NT |  | -0.580^**^ | -0.750^**^ | -0.749^**^ |
|  |  | (0.280) | (0.320) | (0.320) |
|  |  |  |  |  |
| ACT |  | -0.144 | -0.134 | -0.134 |
|  |  | (0.230) | (0.233) | (0.233) |
|  |  |  |  |  |
|  |  |  |  |  |
| Survey wave (1 = 2001, 21 = 2021)=2 |  | -0.249^***^ | 0.000 | 0.000 |
|  |  | (0.072) | (.) | (.) |
|  |  |  |  |  |
| Survey wave (1 = 2001, 21 = 2021)=3 |  | -0.167^**^ | 0.059 | 0.059 |
|  |  | (0.075) | (0.089) | (0.089) |
|  |  |  |  |  |
| Survey wave (1 = 2001, 21 = 2021)=4 |  | -0.083 | 0.132 | 0.133 |
|  |  | (0.078) | (0.094) | (0.094) |
|  |  |  |  |  |
| Survey wave (1 = 2001, 21 = 2021)=5 |  | -0.193^**^ | -0.048 | -0.047 |
|  |  | (0.085) | (0.098) | (0.098) |
|  |  |  |  |  |
| Survey wave (1 = 2001, 21 = 2021)=6 |  | -0.135^*^ | 0.004 | 0.005 |
|  |  | (0.080) | (0.095) | (0.095) |
|  |  |  |  |  |
| Survey wave (1 = 2001, 21 = 2021)=7 |  | 0.010 | 0.149 | 0.151 |
|  |  | (0.094) | (0.109) | (0.109) |
|  |  |  |  |  |
| Survey wave (1 = 2001, 21 = 2021)=8 |  | 0.240^**^ | 0.363^***^ | 0.366^***^ |
|  |  | (0.101) | (0.113) | (0.113) |
|  |  |  |  |  |
| Survey wave (1 = 2001, 21 = 2021)=9 |  | 0.305^***^ | 0.392^***^ | 0.395^***^ |
|  |  | (0.085) | (0.095) | (0.096) |
|  |  |  |  |  |
| Survey wave (1 = 2001, 21 = 2021)=10 |  | 0.295^***^ | 0.352^***^ | 0.356^***^ |
|  |  | (0.085) | (0.096) | (0.097) |
|  |  |  |  |  |
| Survey wave (1 = 2001, 21 = 2021)=11 |  | 0.454^***^ | 0.425^***^ | 0.429^***^ |
|  |  | (0.077) | (0.089) | (0.089) |
|  |  |  |  |  |
| Survey wave (1 = 2001, 21 = 2021)=12 |  | 0.477^***^ | 0.404^***^ | 0.408^***^ |
|  |  | (0.083) | (0.095) | (0.095) |
|  |  |  |  |  |
| Survey wave (1 = 2001, 21 = 2021)=13 |  | 0.622^***^ | 0.538^***^ | 0.542^***^ |
|  |  | (0.079) | (0.092) | (0.092) |
|  |  |  |  |  |
| Survey wave (1 = 2001, 21 = 2021)=14 |  | 0.698^***^ | 0.545^***^ | 0.549^***^ |
|  |  | (0.079) | (0.092) | (0.092) |
|  |  |  |  |  |
| Survey wave (1 = 2001, 21 = 2021)=15 |  | 0.623^***^ | 0.474^***^ | 0.478^***^ |
|  |  | (0.082) | (0.094) | (0.094) |
|  |  |  |  |  |
| Survey wave (1 = 2001, 21 = 2021)=16 |  | 0.637^***^ | 0.498^***^ | 0.502^***^ |
|  |  | (0.078) | (0.091) | (0.092) |
|  |  |  |  |  |
| Survey wave (1 = 2001, 21 = 2021)=17 |  | 0.620^***^ | 0.478^***^ | 0.483^***^ |
|  |  | (0.086) | (0.099) | (0.100) |
|  |  |  |  |  |
| Survey wave (1 = 2001, 21 = 2021)=18 |  | 0.813^***^ | 0.652^***^ | 0.656^***^ |
|  |  | (0.078) | (0.092) | (0.092) |
|  |  |  |  |  |
| Survey wave (1 = 2001, 21 = 2021)=19 |  | 0.987^***^ | 0.803^***^ | 0.808^***^ |
|  |  | (0.080) | (0.092) | (0.093) |
|  |  |  |  |  |
| Survey wave (1 = 2001, 21 = 2021)=20 |  | 0.738^***^ | 0.457^***^ | 0.286^*^ |
|  |  | (0.085) | (0.096) | (0.159) |
|  |  |  |  |  |
| Survey wave (1 = 2001, 21 = 2021)=21 |  | 0.911^***^ | 0.651^***^ | 0.664^***^ |
|  |  | (0.084) | (0.097) | (0.147) |
|  |  |  |  |  |
| Lives in major city (1=Yes)=0 |  | 0.000 | 0.000 | 0.000 |
|  |  | (.) | (.) | (.) |
|  |  |  |  |  |
| Lives in major city (1=Yes)=1 |  | -0.033 | -0.032 | -0.032 |
|  |  | (0.055) | (0.057) | (0.057) |
|  |  |  |  |  |
| [2] Unemployed |  |  | 0.727^***^ | 0.727^***^ |
|  |  |  | (0.062) | (0.062) |
|  |  |  |  |  |
| [3] Not in the labour force |  |  | 0.332^***^ | 0.332^***^ |
|  |  |  | (0.055) | (0.055) |
|  |  |  |  |  |
|  |  |  |  |  |
| Rents current dwelling (1=Yes)=1 |  |  | 0.762^***^ | 0.762^***^ |
|  |  |  | (0.058) | (0.058) |
|  |  |  |  |  |
|  |  |  |  |  |
| Receives welfare/transfer income (1=Yes)=1 |  |  | 0.719^***^ | 0.718^***^ |
|  |  |  | (0.051) | (0.051) |
|  |  |  |  |  |
|  |  |  |  |  |
| Self-assessed health (1=poor ... 5=excellent)=2 |  |  | -0.479^***^ | -0.479^***^ |
|  |  |  | (0.088) | (0.088) |
|  |  |  |  |  |
| Self-assessed health (1=poor ... 5=excellent)=3 |  |  | -1.064^***^ | -1.064^***^ |
|  |  |  | (0.093) | (0.093) |
|  |  |  |  |  |
| Self-assessed health (1=poor ... 5=excellent)=4 |  |  | -1.613^***^ | -1.613^***^ |
|  |  |  | (0.101) | (0.101) |
|  |  |  |  |  |
| Self-assessed health (1=poor ... 5=excellent)=5 |  |  | -1.892^***^ | -1.891^***^ |
|  |  |  | (0.117) | (0.117) |
|  |  |  |  |  |
|  |  |  |  |  |
| COVID year: 2020 (wave 20)=1 |  |  |  | 0.000 |
|  |  |  |  | (.) |
|  |  |  |  |  |
|  |  |  |  |  |
| COVID year: 2021 (wave 21)=1 |  |  |  | 0.000 |
|  |  |  |  | (.) |
|  |  |  |  |  |
|  |  |  |  | (.) |
|  |  |  |  |  |
| COVID year: 2020 (wave 20)=1 # Equivalised household disposable income (annual) |  |  |  | 0.000 |
|  |  |  |  | (0.000) |
|  |  |  |  |  |
|  |  |  |  | (.) |
|  |  |  |  |  |
| COVID year: 2021 (wave 21)=1 # Equivalised household disposable income (annual) |  |  |  | -0.000 |
|  |  |  |  | (0.000) |
|  |  |  |  |  |
| Constant | -2.133^***^ | -2.462^***^ | -2.584^***^ | -2.579^***^ |
|  | (0.046) | (0.206) | (0.243) | (0.243) |
| Observations | 273,943 | 216,425 | 204,930 | 204,930 |
| N_clusters | 31,395 | 24,702 | 23,855 | 23,855 |
| Pseudo R-sq | 0.043 | 0.121 | 0.192 | 0.192 |

Weighted by hhwtsc (SCQ weight). Standard errors clustered on xwaveid. Sample restricted to equiv. income in [0, 300000]. Dependent variable: mealskip (1 = skipped a meal due to money shortage in past 12 months).

^*^ *p* < 0.10, ^**^ *p* < 0.05, ^***^ *p* < 0.01
