# Supplementary material for: Income, food expenditure shares, and severe food insecurity in Australia across 21 waves of HILDA
Source: Health Promot Int. 2026 Jun 4;41(3):daag079. doi: 10.1093/heapro/daag079 (PMC13234612; doi:10.1093/heapro/daag079)
Supplement: daag079_Supplementary_Data [file daag079_supplementary_data.zip › tab_s7_engel_curve_v2_ACCEPTED.docx]

Table S7: OLS — Food Expenditure Share (Engel Curve)

|  | (1) | (2) | (3) | (4) |
| --- | --- | --- | --- | --- |
|  | Bivariate (W21) | Demographics (W21) | Month FEs (W21) | Pooled (all waves) |
| Log equivalised household income | -14.571^***^ | -15.716^***^ | -15.712^***^ | -16.566^***^ |
|  | (0.275) | (0.256) | (0.256) | (0.146) |
|  |  |  |  |  |
| HF Number of in-scope persons in household |  | 3.444^***^ | 3.391^***^ | 2.633^***^ |
|  |  | (0.427) | (0.422) | (0.188) |
|  |  |  |  |  |
| DV: Number of persons aged 15+ years at June 30 2001 |  | 0.723 | 0.762 | 2.021^***^ |
|  |  | (0.508) | (0.502) | (0.259) |
|  |  |  |  |  |
| DV: Number of dependent children aged 0-4 (includes partner's children) |  | -1.578^***^ | -1.542^***^ | -0.576^***^ |
|  |  | (0.468) | (0.465) | (0.197) |
|  |  |  |  |  |
| DV: Number of dependent children aged 5-9 (includes partner's children) |  | -0.647 | -0.611 | 0.834^***^ |
|  |  | (0.477) | (0.472) | (0.200) |
|  |  |  |  |  |
| DV: Number of dependent children aged 10-14 (includes partner's children) |  | 1.000^**^ | 1.031^**^ | 1.879^***^ |
|  |  | (0.502) | (0.497) | (0.202) |
|  |  |  |  |  |
| DV: Number of dependent children aged 15-24 (includes partner's children) |  | 0.459 | 0.476 | 0.908^***^ |
|  |  | (0.319) | (0.319) | (0.157) |
|  |  |  |  |  |
|  |  | (.) | (.) | (.) |
|  |  |  |  |  |
| Lives in major city (1=Yes)=1 |  | 0.448^**^ | 0.442^**^ | 0.050 |
|  |  | (0.211) | (0.211) | (0.122) |
|  |  |  |  |  |
| Constant | 178.285^***^ | 179.197^***^ | 181.621^***^ | 180.600^***^ |
|  | (3.080) | (2.745) | (2.965) | (1.380) |
| Observations | 15,808 | 15,801 | 15,801 | 232,985 |
| R-squared | 0.331 | 0.520 | 0.521 | 0.504 |
| N_clusters | 15,808 | 15,801 | 15,801 | 31,366 |

Weighted by hhwtrp (PQ weight). Standard errors clustered on xwaveid. Dependent variable: food expenditure share (%). Sample restricted to share in [0,100] and income in (0, 300000].

^*^ *p* < 0.10, ^**^ *p* < 0.05, ^***^ *p* < 0.01
