# Supplementary material for: Income, food expenditure shares, and severe food insecurity in Australia across 21 waves of HILDA
Source: Health Promot Int. 2026 Jun 4;41(3):daag079. doi: 10.1093/heapro/daag079 (PMC13234612; doi:10.1093/heapro/daag079)
Supplement: daag079_Supplementary_Data [file daag079_supplementary_data.zip › tab_s8_summary_stats_v2.docx]

Table S8: Summary Statistics — Wave 21 (2021)

Notes: Weighted estimates. SCQ-based variables weighted by hhwtsc; PQ-based variables weighted by hhwtrp. Unweighted N shown in parentheses. Income reported as median (IQR).

| **Variable** | **National** | **NSW** | **Illawarra Shoalhaven** |
| --- | --- | --- | --- |
| Unweighted N | 14629 | 4122 | 277 |
| Meal-skip prevalence (%, hhwtsc) | 3.37 | 2.99 | 4.38 |
| Median equiv. household income ($, hhwtrp) | $ 56,571 | $ 56,310 | $ 56,661 |
| Income IQR (p25–p75) | $ 37,799 – $ 78,786 | $ 37,253 – $ 79,592 | $ 42,253 – $ 72,348 |
| Mean age (hhwtrp) | 46.5 | 46.4 | 48.0 |
| Female (%, hhwtrp) | 50.9 | 51.1 | 47.7 |
| Partnered (%, hhwtrp) | 58.7 | 58.8 | 63.2 |
| Renter (%, hhwtrp) | 27.3 | 28.8 | 25.6 |
| Welfare receipt (%, hhwtrp) | 39.6 | 42.0 | 36.9 |
| Urban (%, hhwtrp) | 72.5 | 76.0 | 64.8 |
| Indigenous (%, hhwtrp) | 3.5 | 3.4 | 1.5 |
| Post-secondary education (%, hhwtrp) | 62.4 | 63.2 | 64.9 |
| BMI: Underweight (%, hhwtsc) | 2.1 | 2.3 | 2.5 |
| BMI: Normal (%, hhwtsc) | 36.1 | 38.5 | 30.3 |
| BMI: Overweight (%, hhwtsc) | 33.9 | 35.2 | 35.9 |
| BMI: Obese I (%, hhwtsc) | 16.4 | 14.2 | 20.9 |
| BMI: Obese II (%, hhwtsc) | 6.6 | 6.0 | 5.9 |
| BMI: Obese III (%, hhwtsc) | 4.8 | 3.6 | 4.6 |
| Self-assessed health: Excellent (%, hhwtsc) | 11.5 | 12.8 | 11.5 |
| Self-assessed health: Very good (%, hhwtsc) | 35.4 | 35.6 | 38.0 |
| Self-assessed health: Good (%, hhwtsc) | 36.1 | 35.3 | 36.2 |
| Self-assessed health: Fair (%, hhwtsc) | 13.8 | 12.9 | 11.2 |
| Self-assessed health: Poor (%, hhwtsc) | 3.3 | 3.3 | 3.1 |
| Mean K10 distress (hhwtsc) | 17.9 | 17.9 | 16.6 |
